# Supplementary material for: What does it mean to have experienced the death of a relative in a context of social and funeral restrictions? Lessons from the pandemic for bereavement research and clinical practice
Source: PLoS One. 2025 Sep 19;20(9):e0331946. doi: 10.1371/journal.pone.0331946 (PMC12448364; doi:10.1371/journal.pone.0331946)
Supplement: S1 Positioning — (DOCX) [file pone.0331946.s002.docx]

# Researchers’ background and subjective positioning on the topic

CB : CB is a clinical psychologist and a doctoral student. Before starting her thesis, she worked as a hospital psychologist with chronically ill children and adolescents. In her clinical practice, she has been confronted with the issue of grief in number of ways, working with patients and families bereaved by death or other types of loss. Working during the pandemic, she was confronted with social restrictions in medical care and end-of-life situations. Personally, she experienced the death of a relative during the pandemic and was only able to say a limited farewell, at a distance from the rest of her family. Her professional and personal experiences inspired her to research on the subject. Through her experiences, she learned how differently people can react to these experiences and how diverse the needs of people in such situations are, which guided the aim of the current study.

EZ : She is professor in clinical psychology and person-centered therapy. She also retains a clinical practice with bereaved and traumatized clients. From early childhood, she has experienced the loss of significant people and pets through death and separation. This led her to tame death by doing an internship in a palliative care unit and she started conduct research on bereavement with renown scientists in the field. She did not experience the death of a close relative during the pandemic. From March 2020, she was concerned that the media and experts would induce by their warnings a potential self-fulfilling prophecy about a “pandemic of pathological grief”, without considering the vast ressources that humans show in dealing with adversity.

LA : She is a doctoral student in family sciences. Her practice as a clinical nurse includes supporting bereaved people, teaching courses at the University of Quebec in Outaouais and coordinating the CE Deuil research laboratory. Her interest in grief began at a very young age. Having experienced loss as a young child and being the child of two orphaned parents for whom mourning was taboo, the desire to talk about death and mourning proved essential to her practice. Thus, this article focusing on pandemic mourning from an innovative angle allows us to revisit the realities of bereaved people and to review the relationship with rituals.

CV : She is a professor of nursing and family sciences. She’s been in clinical practice with bereaved people for over thirty years, especially in the context of perinatal and parental death. On a personal side, she had parents that became orphans at a very young age. This personal experience led the way for her to pursue the understanding of the grieving process, specifically the prolonged grief over time. She did not personally experience bereavement during the pandemic, but she led many support groups during this period. She noted that this global period did not necessarily have a negative effect on those that suffered bereavement. They realized the importance of grief in their lives and the role that rituals must play after the death of a loved one.
